# A systems biology approach for discovering the cellular and molecular aspects of psychogenic non-epileptic seizure

**DOI:** 10.3389/fpsyt.2023.1116892

**Published:** 2023-05-12

**Authors:** Mahdi Malekpour, Aida Jafari, Mohammad Kashkooli, Seyed Reza Salarikia, Manica Negahdaripour

**Affiliations:** ^1^Student Research Committee, Shiraz University of Medical Sciences, Shiraz, Iran; ^2^Pharmaceutical Sciences Research Center, Shiraz University of Medical Science, Shiraz, Iran; ^3^Department of Pharmaceutical Biotechnology, School of Pharmacy, Shiraz University of Medical Sciences, Shiraz, Iran

**Keywords:** PNES, psychogenic non-epileptic seizure, systems biology, molecular pathogenesis, epilepsy, functional seizure

## Abstract

**Objectives:**

Psychogenic non-epileptic seizure (PNES) is the most common non-epileptic disorder in patients referring to epilepsy centers. Contrary to common beliefs about the disease’s harmlessness, the death rate of PNES patients is similar to drug-resistant epilepsy. Meanwhile, the molecular pathomechanism of PNES is unknown with very limited related research. Thus, the aim of this *in silico* study was to find different proteins and hormones associated with PNES via a systems biology approach.

**Methods:**

Different bioinformatics databases and literature review were used to find proteins associated with PNES. The protein-hormone interaction network of PNES was constructed to discover its most influential compartments. The pathways associated with PNES pathomechanism were found by enrichment analysis of the identified proteins. Besides, the relationship between PNES-related molecules and psychiatric diseases was discovered, and the brain regions that could express altered levels of blood proteins were discovered.

**Results:**

Eight genes and three hormones were found associated with PNES through the review process. Proopiomelanocortin (POMC), neuropeptide Y (NPY), cortisol, norepinephrine, and brain-derived neurotrophic factor (BDNF) were identified to have a high impact on the disease pathogenesis network. Moreover, activation of Janus kinase-signaling transducer and activator of transcription (JAK–STAT) and JAK, as well as signaling of growth hormone receptor, phosphatidylinositol 3-kinase /protein kinase B (PI3K/AKT), and neurotrophin were found associated with PNES molecular mechanism. Several psychiatric diseases such as depression, schizophrenia, and alcohol-related disorders were shown to be associated with PNES predominantly through signaling molecules.

**Significance:**

This study was the first to gather the biochemicals associated with PNES. Multiple components and pathways and several psychiatric diseases associated with PNES, and some brain regions that could be altered during PNES were suggested, which should be confirmed in further studies. Altogether, these findings could be used in future molecular research on PNES patients.

## 1. Introduction

Psychogenic non-epileptic seizure (PNES) is a kind of seizure with identical characteristics to epileptic seizures. PNES is recognized by sudden changes in emotions, actions, behaviors, and alertness (1). PNES patients have no abnormal electrical discharge during a seizure (2), while patients with neurological seizures have abnormal electrical activity that could be measured by electroencephalography (EEG) (3). This abnormal electric activity in the brain cells causes convulsions and loss of consciousness, memory, muscle control, and movements (3).

PNES is the most common non-epileptic disorder in patients referring to epilepsy centers (4). A systematic review by Asadi-Pooya and colleagues revealed the estimated incidence of PNES based on qualitative analysis about 3.1 persons per 100,000 population per year and prevalence was about 108.5 persons per 100,000 population, in the United State (5). PNES patients account for over 20% of patients among all refractory seizure disorders referred to any epilepsy clinics each year, and only around 4% of cases are known as true epilepsy annually (6). PNES, similar to other functional disorders, is more common in women than in men (7). PNES is estimated to be three times more prevalent in women (8). This difference between men and women may be caused by some early social life events, such as childhood physical and sexual abuse, inherent brain connection problems, or differences in susceptibility to physical or emotional traumas between men and women (7). PNES occurs more commonly in the adult group, but it can also be detected in children (9). Childhood environmental psychological traumas are one of the major risk factors of PNES (9).

In a study done on 5,508 patients in 2020, consisting of 674 diagnosed PNES patients, it was shown that PNES patients had a 2.5 times higher Standardized Mortality Ratio (SMR) than the whole population, death at a rate similar to individuals suffering from drug-resistant epilepsy (10). Epilepsy was the most common distinguished cause of death (23.6%), followed by neoplasia (14.5%), suicide (7.3%), and accidental poisoning (9.1%) (10). Thus, it is important to reduce the morbidity and mortality of PNES through early disease diagnosis and effective therapeutics such as psychological therapies, anti-depressant drugs, and so on (11).

Despite all the studies done on PNES, its diagnosis is still challenging and might even take years to determine it definitely (12). Physicians need to consider PNES as a differential diagnosis for any patients with seizure-like symptoms (12). Therefore, finding any biological markers to differentiate between PNES patients and epileptic seizures could be helpful.

As a psychological disease, it has an important impact on patients’ lives. Depression and its symptoms are common comorbidities associated with PNES (13).

While a lot of research has been done on PNES environmental risk factors, little is known about its molecular predispositions (14). Previous studies showed that systems biology approaches have advantages in clarifying the cellular and molecular aspects of diseases using omics datasets (15–17). Additionally, one of the applicable ways to find pathways relevant to diseases, is gathering different genes from different databases and analyzing them with network science tools to find new shreds of evidence for the pathomechanism of the diseases (18, 19).

This research aims to study PNES through systems biology tools to see the role of molecular and cellular aspects in the pathogenesis of the disease and its comorbid situations according to previous studies.

## 2. Materials and methods

### 2.1. Finding associated genes and hormones

According to the fact that few high and low-throughput experiments have been done on PNES disease, few genes have been reported to be associated with PNES. Genes and proteins related to PNES were found with the help of different databases, text-mining tools, and also with literature reviews. “PNES,” “psychogenic nonepileptic seizure,” “psychogenic seizure,” “functional seizure,” and “dissociative seizure” were searched in Online Mendelian Inheritance in Man database (OMIM; omim.org) (20), DisGeNET^1^ (21, 22), Geneshot (maayanlab.cloud/geneshot) (23), ClinVar^2^ (24), GENE part of NCBI^3^ (25), and Orphanet^4^ (26) to find the associated genes. Then, the suggested genes were manually rechecked with the references and original studies provided by databases. If no publication supported the association of PNES and a gene, the name of the gene or its full name with PNES was searched in PubMed and Google Scholar to find evidence for the association of the gene with PNES. If no evidence was found for the gene, it was excluded from the study. On the other hand, “PNES,” “psychogenic nonepileptic seizure,” “psychogenic seizure,” “non epileptic seizure,” “functional seizure,” “dissociative seizure,” or “pseudoseizure” were searched in titles of Google Scholar, and these terms with the terms such as “biomarker,” “polymorphism,” “gene,” “hormone,” “protein,” “SNP,” “marker,” and “serum level” were searched in PubMed to find other associated genes and hormones and small molecules associated with PNES. On the next step, the title and abstract of these publications were reviewed, and other associated genes and hormones were found. At the end, by retrieving the main text of articles and reviewing the full text of articles, the suggested hormones and genes were validated.

### 2.2. Protein-hormone interaction network reconstruction

For reconstruction of the protein-hormone network of PNES, the PNES-associated genes and hormones were imported to the multiple identifier section of the STITCH database^5^ (27). STITCH is a database that can be used to get a clear and comprehensive view of the relationship between different molecules and proteins in the body with multiple pieces of evidence. STITCH database automatically adds some chemicals and proteins to the network based on various databases and methods to get the connections between their biochemicals. The added nodes could be the missing nodes of the network.

After the construction of the protein-hormone interaction network, the network was imported to the Gephi software (28), and an undirected unweighted network was reconstructed in Gephi. The giant component filter of Gephi was used for removing the outgroup genes, and then, the centrality measures of the network were computed. The centrality measures were used for finding the hub genes of the network. Then, the network was visualized based on increasing the size of the nodes according to betweenness centrality and darkening the node colors according to eigenvector centrality.

### 2.3. Functional enrichment analysis

Enrichment analysis is a process through which new and more understandable information and knowledge can be obtained by comparing newly obtained gene and protein sets with the previously classified sets.

For the enrichment analysis, the result of the STITCH database was imported to the Enrichr (maayanlab.cloud/Enrichr) database (29–31) to find the associated pathways of the Kyoto encyclopedia of genes and genomes (KEGG; genome.jp/kegg) (32–34) and the biological processes of gene ontology (GO; geneontology.org) (35, 36) database with the PNES gene set.

### 2.4. Gene-psychiatric disease association network construction

Due to several comorbidities of psychological diseases with PNES, finding the molecular mechanism of the association of psychological diseases to PNES is very important. Psychiatric disorders Gene association NETwork (PsyGeNet; psygenet.org) database (37) is an appropriate database to find the associations between genes and psychological diseases. This database automatically extracts information from the literature using the text mining tool curated by experts. The database has information on major categories of depression, bipolar disorder, alcohol use disorders, cocaine abuse disorders, schizophrenia, substance-induced depressive disorder, psychosis, and cannabis abuse disorder.

The PsyGeNet2r R package was used to find the comorbidities of the PNES-associated genes and psychiatric disorders. To find the association of genes with the major classes of psychiatric disorders, a heatmap was drawn. In addition, a Panther graphic was drawn to illustrate the class of proteins related to each psychiatric disorder class. In the next step, the associated genes of PNES were used for plotting a network chart of genes and their associated psychiatric diseases. Furthermore, a heatmap of the association of genes to each psychiatric disorder was drawn according to the evidence index of association. This heatmap was drawn to confirm the validation of the associations between PNES genes and psychiatric disorders.

### 2.5. Biomarker secretion region

During the literature reviews, type of the association of each protein with the PNES was found in different articles. Some of the protein concentration levels are altered in the blood during the PNES disease course. The source of altered protein secretion levels in the blood and especially the brain sources could help to discover the region affected by PNES.

Those proteins with an altered concentration in blood were searched in The Human Protein Atlas Database (38) available on www.proteinatlas.org. After finding the section of each protein in the tissue tab, the image of the protein expression overview of each protein was downloaded, and the details about the specific cells that express each tissue were collected. This collected data can help to discover the regions that express proteins in the body and brain.

## 3. Results

### 3.1. Finding PNES-associated genes and hormones

As expected, there were few genes, proteins, and hormones reported in previous studies that were associated with PNES. Moreover, various biochemicals were found in the search process, which could not be used in our study due to the lack of a healthy control group. The available studies compared PNES gene expression with other diseases. DisGeNET only found two genes, the GENE section of NCBI discovered five genes, and OMIM also proposed another gene. While databases such as ClinVar and Orphanet did not report any genes. However, Geneshot found 87 unique genes. When these genes were manually checked, many of them did not have enough evidence for being associated with PNES. Their names were only mentioned in the articles wherein PNES was also mentioned. On the other hand, 56 articles were found in PubMed based on title and abstract, and 537 related publications were identified in Google Scholar. By reviewing the title and abstracts of these articles, some other biochemicals were discovered, which are presented in the Supplementary Table. The validated biochemicals are also mentioned in Table 1 (Supplementary Table 1).

**Table 1:** The associated proteins and hormones to PNES with the type of association and articles showing the associations.

Gene name	Type of association	PMID
*BDNF*	Altered level	20921514
*S100B*	Altered level	30253122
*NPY*	Altered level	30929648
*GH1*	Altered level	2716948
*GABRA5*	Polymorphism	32938993
*NSD1*	Polymorphism	32938993
*FAAH2*	Polymorphism	25885783
*PRL*	Altered level	28927333
Hormone Name		
Norepinephrine	Altered level	2716948
Cortisol	Altered level	2,716,948
ACTH	Altered level	30929648

### 3.2. Protein-hormone interaction network

**Figure 1:**
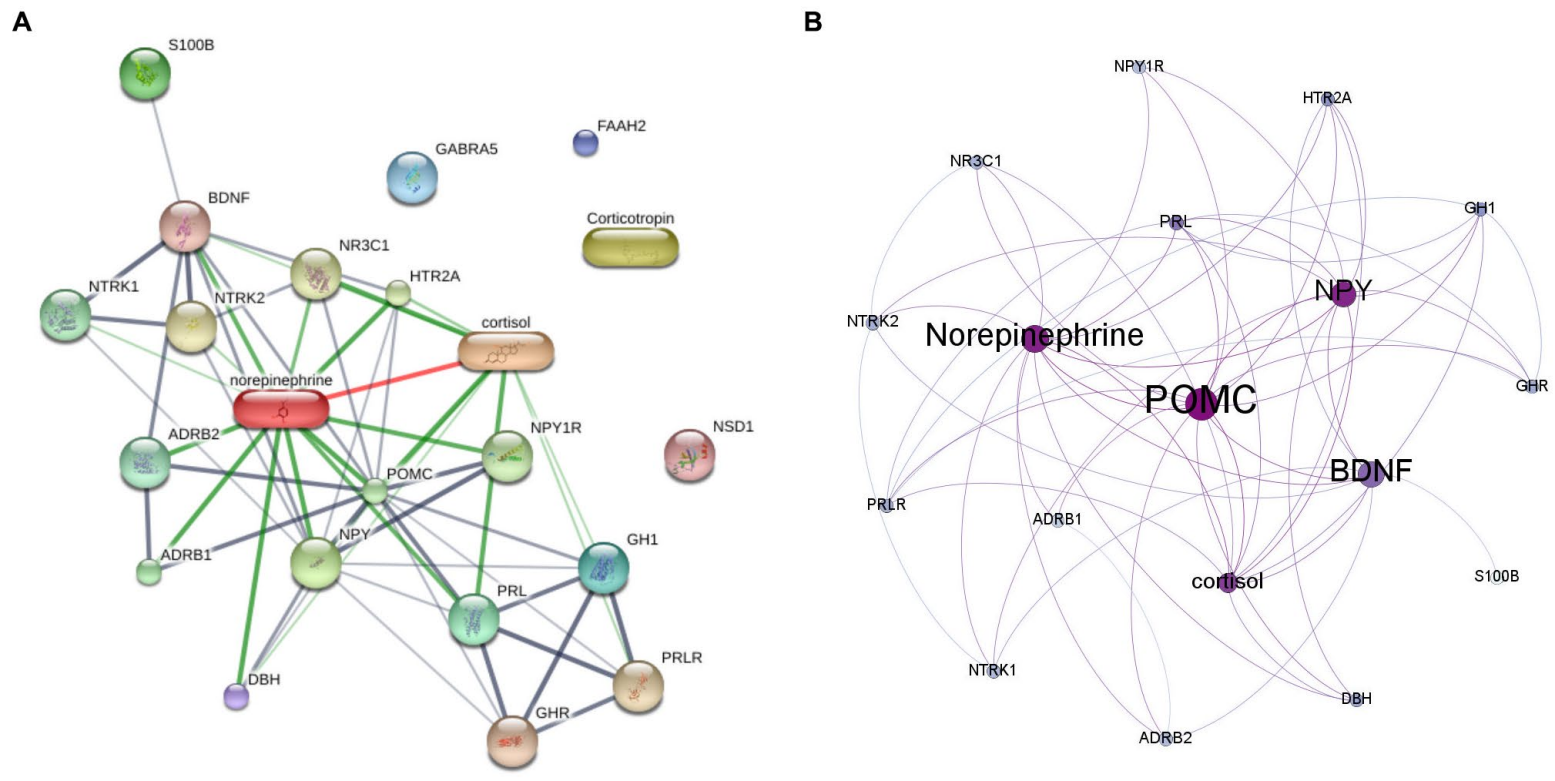
The protein hormone interaction networks. **(A)** The protein-hormone interaction generated by STITCH database. **(B)** The result of centrality measurment analysis of protein-hormone interaction network by Gephi software. The size of the nodes represents the betweenness centrality, and the darkening of the nodes represents the eigencentrality of each node.

As shown in Figure 1A, the protein-hormone interaction network of the associated biochemicals with PNES was reconstructed by the STITCH database. Then, to measure the centrality parameters of these genes (nodes), the network was reconstructed in the Gephi software. As presented in Table 2, proopiomelanocortin (POMC), neuropeptide Y (NPY), norepinephrine, and cortisol had the highest degree in the reconstructed network. POMC, NPY, norepinephrine, brain-derived neurotrophic factor (BDNF), and cortisol had the highest betweenness centralities. POMC, NPY, norepinephrine, cortisol, and prolactin (PRL) had the highest eigenvector centralities. In Figure 1B, the reconstructed network can be seen. The size of the nodes increases with the increase in betweenness centrality, and the darkening of the nodes showed an increase in eigenvector centrality (Supplementary Table 2).

**Table 2:** The result of the centrality measurements of the proteins and hormones associated with PNES.

Name	Degree	Eigen centrality	Closeness centrality	Betweenness centrality
POMC	14	0.85	0.911765	1
Norepinephrine	13	0.809524	0.882353	0.929796
BDNF	9	0.68	0.764706	0.696732
NPY	12	0.772727	0.852941	0.920435
Cortisol	10	0.708333	0.794118	0.833853
PRL	7	0.607143	0.696078	0.646203
NTRK2	5	0.566667	0.637255	0.421716
NR3C1	4	0.548387	0.607843	0.403042
ADRB2	4	0.566667	0.617647	0.370222
GH1	6	0.586207	0.666667	0.541524
GHR	5	0.566667	0.637255	0.448099
PRLR	5	0.53125	0.617647	0.438185
ADRB1	3	0.53125	0.578431	0.291599
HTR2A	5	0.586207	0.647059	0.553783
DBH	4	0.548387	0.607843	0.465274
NPY1R	3	0.53125	0.578431	0.360298
NTRK1	4	0.548387	0.607843	0.376288
S100B	1	0.414634	0.45098	0.088509

### 3.3. Functional enrichment analysis

**Figure 2:**
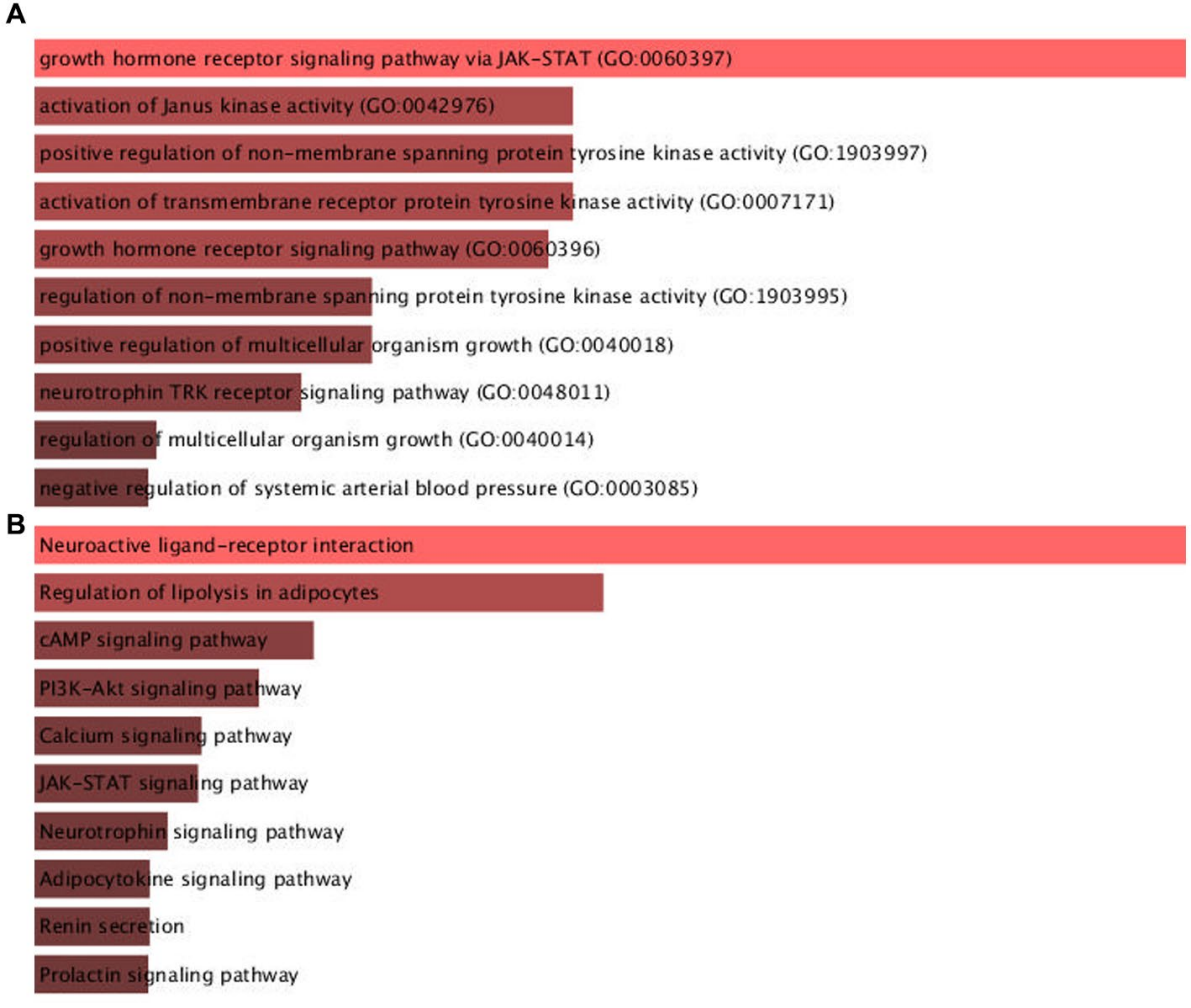
The result of pathways associated to PNES pathogenesis enriched in Enrichr database based on **(A)** GO biological process and **(B)** KEGG databases.

The result of the protein-hormone interaction network was imported to the Enrichr database for enrichment analysis based on KEGG 2021 Human and GO biological process 2021. The most associated pathways with PNES based on the combined score of GO were the growth hormone receptor signaling pathway via Janus kinase-signaling transducer and activator of transcription (JAK–STAT), induction of Janus kinase (JAK) activity, and growth hormone receptor signaling through pathways such as the activation of transmembrane receptor protein kinase, regulation of non-membrane tyrosine kinase activity, and neurotrophin tropomyosin related kinase (TRK) receptor signaling. Besides, the KEGG database suggested the neuroactive ligand-receptor interaction, regulation of lipolysis in adipocytes, phosphatidylinositol 3-kinase (PI3K)/protein kinase B (AKT) signaling, and neurotrophin signaling pathway. The results of the enrichment analysis in detail, such as the contribution of each gene in each pathway, can be seen in Supplementary Table 3. Moreover, the result of the first pathways based on the combined score can be seen in Figure 2 and Supplementary Table 3.

### 3.4. Gene-psychiatric disease association network

**Figure 3:**
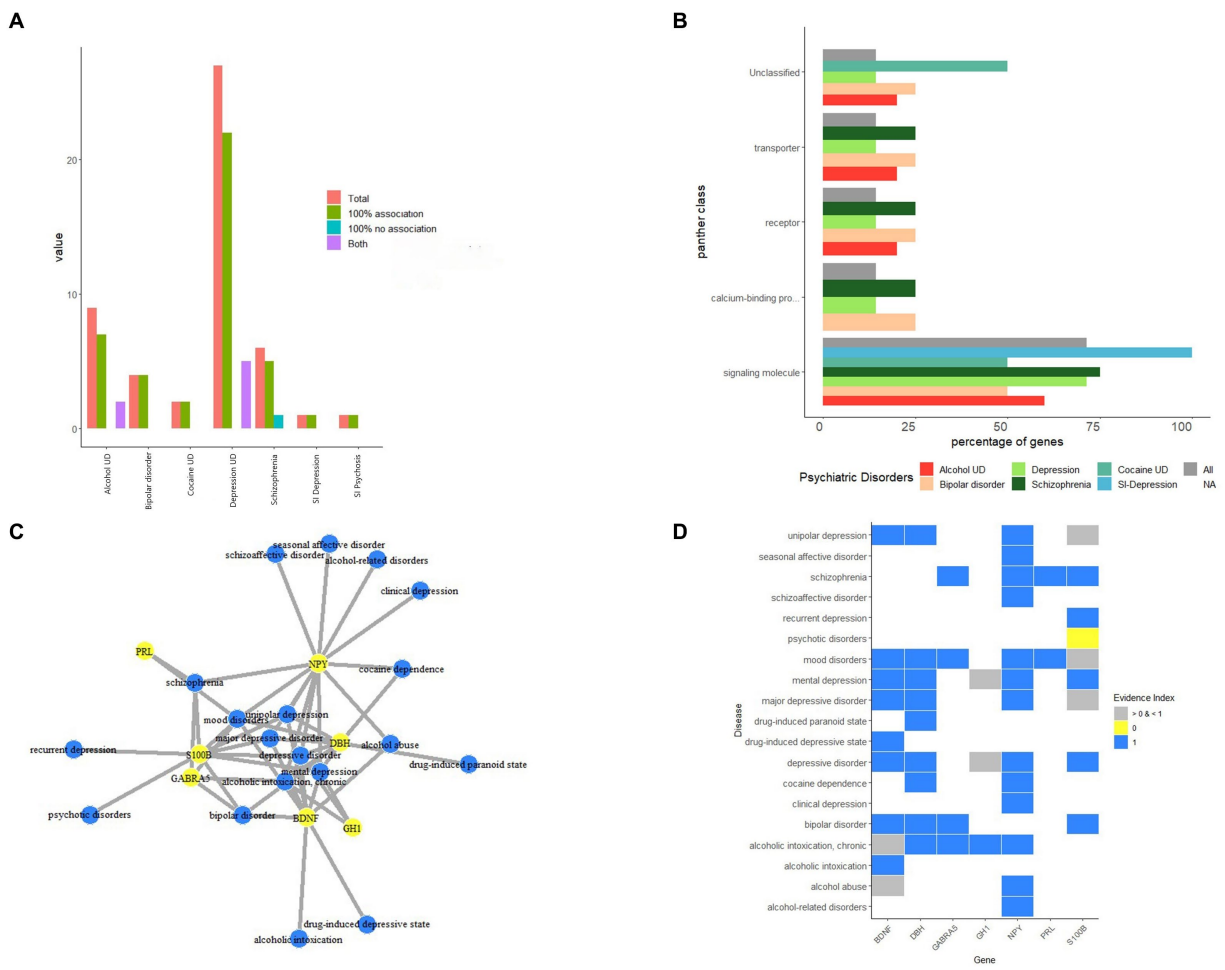
The results protein-psychiatric disease association based on PsyGeNET database. **(A)** The association of associated proteins of PNES to each major psychiatric disease. **(B)** The protein class of proteins associated with PNES. **(C)** The protein-psychiatric disease network. **(D)** The heatmap of evidence showing the association of proteins and psychiatric diseases.

Several reports showed evidence of comorbidities between psychiatric diseases and PNES. Despite the high phenotypic evidence between the psychiatric diseases associated with PNES, there is little molecular evidence of how these illnesses relate to each other. PsyGeNet is a good platform to find the molecular association between these types of diseases. The results are illustrated in Figure 3. The main associated major psychiatric disease category with PNES proteins was depression disorders followed by schizophrenia and alcohol-related disorders. Furthermore, based on the result of the Panther class of the associated genes with PNES, these genes are involved in the major classes of psychiatric diseases via signaling pathways. Based on this analysis, BDNF was mainly associated with different depressive disorders, alcoholic intoxication, and mood disorders, while the evidence provided by the PsyGeNet database was weak for alcoholic intoxication. S100 calcium-binding protein B (S100B) was also related to mood disorders, bipolar disorders, and psychotic disorders, but there is not enough evidence about the association between mood and psychotic disorders. Gamma-aminobutyric acid type A receptor subunit Alpha 5 (GABRA5) was linked to schizophrenia, mood disorders, alcoholic intoxication, and bipolar disorders. PRL was just associated with schizophrenia and mood disorders. NPY was associated with schizophrenic disorders, depressive disorders, mood disorders, and alcohol-related disorders.

### 3.5. Biomarker secretion region

**Figure 4:**
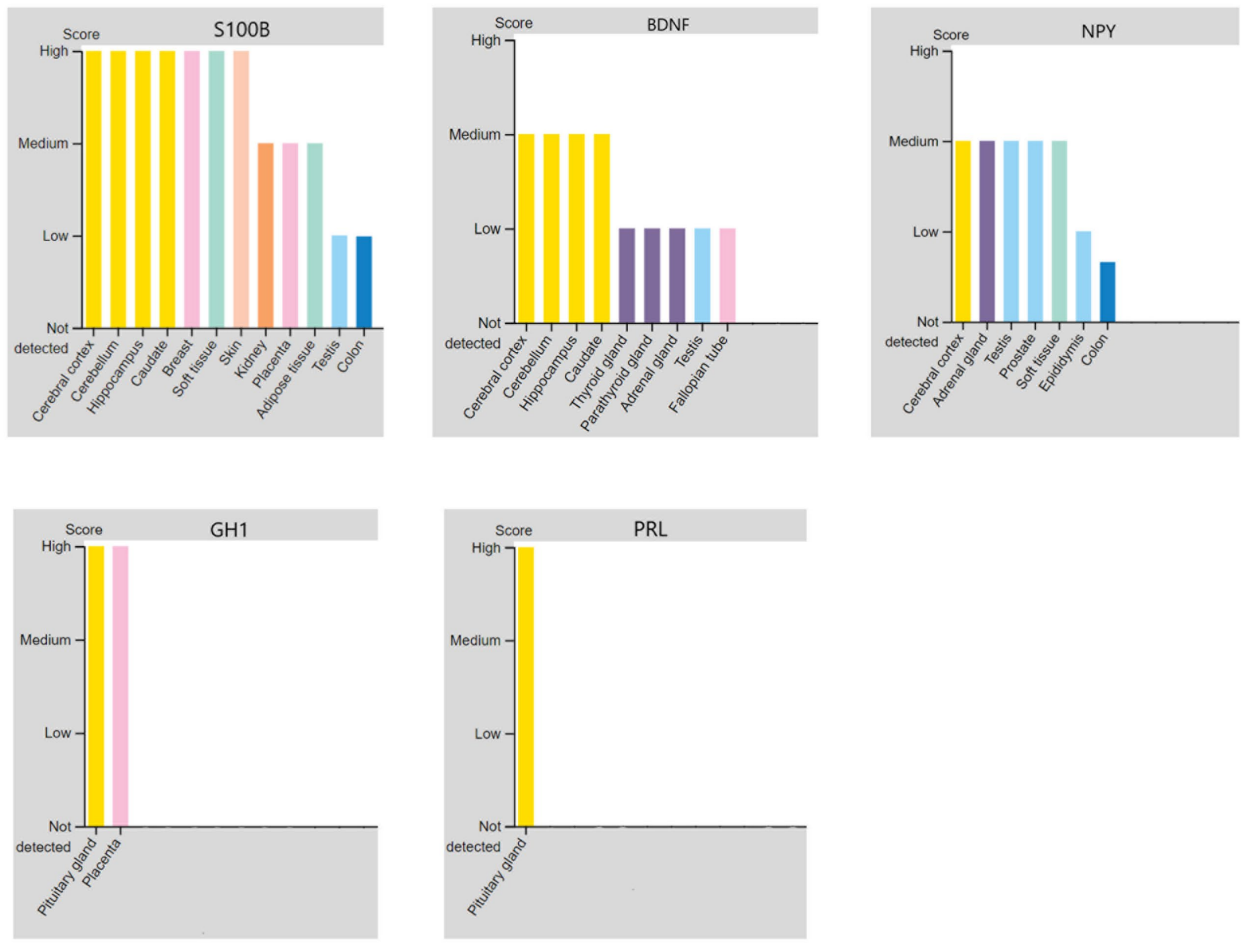
The regions in the brain that could express and secrete the altered proteins of PNES in blood based on the protein atlas database.

**Figure 5:**
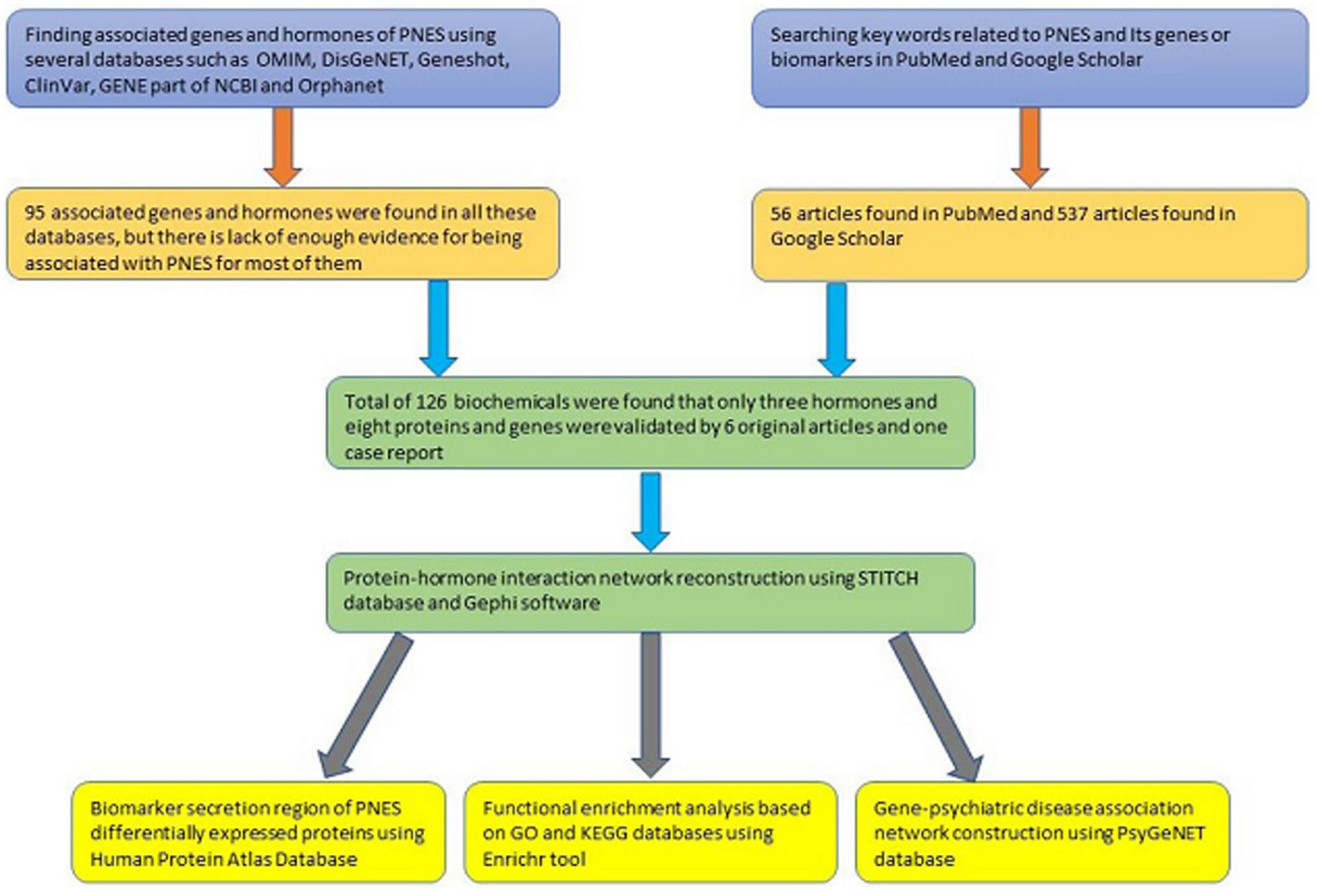
The step-by-step workflow of this study and the number of articles found in each step.

Because there is limited evidence about the molecular pathomechanism of PNES, and as the involved regions of the brain in PNES have not been found yet, discovering regions that secrete specific biomarkers can be helpful for further studies. During the literature review process, BDNF (39), growth hormone 1 (GH1) (40), NPY (41, 42), PRL (41), and S100B (43) were found with altered concentrations in the blood. Among all of them, just S100B’s concentration was increased in the blood (43). The results of the protein expression levels in different tissues are shown in Figure 4, and the details about the expression level of proteins in each type of cell in tissues can be found in the Supplementary Table 4. Additionally, Figure 5 shows a step-by-step workflow of this study.

## 4. Discussion

PNES is a kind of seizure that was previously thought to be a pseudoseizure caused by psychological problems. However, nowadays the border between psychological and organic problems is disappearing. Currently, many brain structural and neurobiological signs related to PNES have been found (44). In this article, our aim was to investigate the location of the involvement in the brain, the relationship between PNES and other mental illnesses, and the most important proteins, hormones, and molecular pathways involved in the pathophysiology of the disease based on a systems biology approach.

### 4.1. Hub genes and hormones

Finding the hub genes that regulate the pathogenesis of the diseases can help to find the exact pathomechanisms of the diseases and also discover new treatments for these diseases.

POMC was the first important gene in the gene-hormone interaction network. POMC was not a definite finding of our literature review, but the STITCH database suggested the gene to be involved in the gene-hormone interaction network. POMC, which is a prohormone of ACTH, is mainly produced in the neurons of the pituitary and hypothalamus (45). In a previous study, the baseline amount of ACTH was greater than the normal group showing the hyperactivity of the hypothalamic–pituitary–adrenal (HPA) axis (42).

Early-life stress (ELS) raises the sensitivity thresholds for stress-related disorders such as severe depression and anxiety, by altering the structure and function of brain circuits and endocrine pathways. One of the circuits that constantly may be activated is the HPA axis. ELS also reduces the methylation of the POMC gene, which is a part of the HPA axis (46). Moreover, an association between ACTH level and the severity of long-term stress, particularly sexual abuse, has been found previously in PNES patients (41). According to these findings, the association between the HPA axis function and PNES should be investigated in further studies.

Norepinephrine is the second hub in the network of protein-hormone interaction based on the eigenvector and betweenness centrality. Previously, it was found that the level of norepinephrine decreased in the PNES patients compared to the normal groups (40). Norepinephrine is both a neurotransmitter and a hormone, which acts as a fight-and-flight response agent via autonomic and noradrenergic systems (47, 48). The brain’s noradrenergic system disruption might have a role in stress-related disease susceptibility. Disruption in the noradrenergic system can cause the over-activation of the HPA axis, which could be a reason for neuropsychological illnesses caused by stress (48). It should be mentioned that norepinephrine, epinephrine, and acetylcholine are the main neurotransmitters of the autonomic system (49). Previously, the association of heart rate variability (HRV) to PNES was found in multiple studies (50–52). HRV is mainly regulated by the autonomic system, and the reason for autonomic system imbalance in PNES patients’ needs further studies.

The third important node in the protein-hormone interaction network was BDNF because of its high betweenness centrality measure and other high centrality measures of the network. BDNF is a neurotrophin protein, which mainly regulates axonal and dendritic growth in several areas of the brain. BDNF also regulates synaptic transmission and plasticity at mature synapses (53, 54).

A previous study found that in PNES patients, the BDNF level of serum would be decreased, which was associated with the concentration of BDNF in the brain (53). In this study, the low level of BDNF was suggested to be because of the high-stress level of the PNES patients (53). Besides, it was found that low levels of BDNF were associated with low drug response in major depressive disorder (MDD) (55). Thus, BDNF and its downstream pathway could probably be suggested as a drug target for depression. Further studies are needed to show the usage of this protein as a target for the treatment of PNES.

The fourth important node was NPY. Previous studies found low levels of NPY in PNES patients (41). Moreover, it was found that the combination of NPY and ACTH could differentiate healthy patients from PNES ones (42). NPY is a neurotransmitter of the sympathetic system, which can affect the secretion of ACTH and as a result the HPA axis (41). Previous studies suggested that lower levels of NPY are associated with stress-related conditions, and its therapeutic role in the management of these diseases was proposed (41). Hence, the strategies that could help to upregulate NPY in the brain and suppress the HPA axis could be considered for treating PNES patients.

Cortisol hormone is the fifth hub component in the gene-protein interaction network. Different studies showed different associations between PNES and cortisol levels in the blood such as increased basal cortisol levels (56, 57). Post-ictal cortisol levels (58) in PNES patients show the involvement of the HPA axis in PNES. In reaction to ACTH, the adrenal cortex releases cortisol, and the increased cortisol level can be measured, which is a component of the HPA axis (59). This increased cortisol level is associated with mental health problems (60, 61). Previous neuroimaging investigations also found impaired stress response circuits in the brain of PNES individuals (62). Thus, finding accurate stress-related mechanisms, especially the HPA axis pathway, is important for treating PNES patients.

### 4.2. Associated pathways to PNES

The most distinguished pathways in the enrichment analysis were JAK–STAT signaling. JAK–STAT signaling pathways contributed to neural development, differentiation, survival, and plasticity. While, the dysregulation of the JAK–STAT signaling could be a sign of inflammation seen in epilepsy, neurodegenerative diseases, and brain cancer and lesions (63). One of the stimulators of JAK–STAT signaling is GH, which activates signaling via tyrosine kinase functioning (64). Previously, it was found that the neuronal plasticity and functional connectivity of the brain is changed in PNES patients, suggesting the biological origin of the disease (65–68). Studying the growth hormone receptor signaling pathway via JAK–STAT signaling could help to better understand the mechanism of the disease.

Pathways related to neurotrophic pathways could also contribute to the neural plasticity of PNES patients. On the other hand, it was previously found that BDNF, which is a neurotrophic hormone, is lower in PNES patients. BDNF contributes to neurotrophic pathways and is essential for the regulation of neuronal growth and plasticity (53, 54). The neurotrophin signaling pathway is mainly started by the activation of two receptors, the nerve growth factor receptor (NGFR) and neurotrophic receptor tyrosine kinases (NTRKs). BDNF activates the NTRK receptors, which was the first enriched GO pathway in our results. Activated NTRK receptors, as well as NGFR signaling, activates the downstream pathways such as mitogen-activated protein signaling (MAPK), RAS, and PI3K/AKT signaling, which results in different happenings in the brain (65). The activation of PI3K/AKT signaling by the neurotrophin signaling pathway leads to the prevention of apoptosis and acts as a cell survival stimulus pathway (69).

Some previous works showed that there are also some relations between different types of epilepsy such as temporal lobe epilepsy (TLE) and psychiatric disorders. It was shown 23% of patients with TLE experience anxiety disorders followed by 17% mood disorders, 13% psychotic disorders, and 3% somatoform disorders (70). Moreover, 9.6% of patients with temporal lobe epilepsy were reported to experience bipolar disorder (71). Several different studies recorded that psychiatric features were often present before the epilepsy diagnosis and even psychiatric diagnosis might occur before and after epilepsy onset with similar frequency (72–74). These data show the necessity of exploring common pathways (75). It has been shown that alterations in BDNF expression could play a role in the development of epilepsy as well as psychiatric disorders, such as bipolar disorder and depression (76, 77). Studies stated that BDNF and its conjugate receptor (i.e., Tropomyosin receptor kinase B or TrkB) are increased in animal models and humans with epilepsy, particularly in temporal and hippocampal areas, and inhibition of BDNF–TrkB signaling and reinforcing the NPY system could be a potential therapeutic strategy for epilepsy (78). In contrast, low levels of BDNF are present in bipolar 1 disorder and could be a biomarker (77). These data suggest that further investigations could find common pathways involved in seizures including TLE, PNES, and psychiatric disorders.

### 4.3. Association of PNES and psychiatric diseases

Previous researchers suggested that PNES patients have more comorbidity than psychiatric disorders such as depression, anxiety, and personality disorders. However, it is not clear if the associations are causal or not (79, 80). One of the reasons suggested was the conversion reactions of psychological diseases that causes PNES (81). However, the molecular association between different psychiatric diseases and PNES was discussed in this study.

Our first finding of the proteins associated with PNES in PsyGeNet was that these proteins were more associated with depressive disorders than other major psychiatric disorders. A systematic review and meta-analysis about the rate of depression in PNES versus epileptic seizure also reported that a higher percentage of PNES patients have depression (82). Our finding could show that there is also a molecular association between depressive disorders and PNES. On the other hand, based on Figure 3B, the associated genes to PNES affect psychiatric diseases via signaling pathways.

Co-occurrence of PNES with many of the psychiatric diseases with the reported molecular relationship was found including schizophrenia (81), alcoholism (83), major depressive disorder (84), bipolar disorders (84), and mood disorders (84). Though the occurrence of PNES is important, the co-occurrence of PNES with psychiatric problems can affect the quality of life of patients, and it should be considered in therapy (85).

### 4.4. Region of PNES occurrence in the brain

PRL is a hormone mainly used in previous research to differentiate epileptic seizure from PNES after happening of the seizure (39). However, in one of the researches, a lower level of the PRL protein was detected in the PNES patients than in the control group (41). PRL is mainly secreted from the pituitary gland. It was previously suggested that decreased levels of prolactin could worsen the onset of schizophrenia symptoms (86) and has a probable role in severe depression development (87).

Our study showed that BDNF is secreted from different parts of the brain, but the main regions for its secretion were found to be the cerebral cortex, cerebellum, and hippocampal complex. Downregulation of the secretion from these parts could be the reason for the lower BDNF levels in the blood. Previous studies also found that brain connectivity was decreased in PNES patients, which could be the reason for the decreased level of BDNF (53, 54).

Growth hormone 1 (GH1) is another protein whose altered level has been detected after the PNES attack (40). There is very little evidence about the association between PNES and GH1, but there is some evidence about the relationship between GH1 and other brain-related diseases. During the process of epileptogenesis, the upregulation of GH was detected (88), and also GH1 downregulation can be a result of epilepsy, which can cause depression (89).

NPY is another neurotransmitter whose downregulation was reported in PNES patients (41, 42). NPY has a medium score of expression in different organs such as the cerebral cortex, adrenal gland, testis, prostate, and soft tissues. In these organs, NPY can be detected mainly in glandular cells, peripheral nerves, Leydig cells, and neutrophils. Previously it was found that depressive and anxiety-related actions can be seen in knock-out mice (90). Moreover, some evidence showed the association of NPY with human depression (91). There should be more studies about the role of NPY-impaired levels in PNES to probably find new solutions for the disease.

S100B is the only protein whose expression level is increased in PNES patients (43). Based on our analysis, S100B can be detected in many tissues, but it is mostly detected in the cerebral cortex, cerebellum, hippocampus, caudate, breast, and soft tissue. S100B could be detected in neuronal cells, glial cells, and also in neutrophil cells. Besides, it is expressed in the peripheral nerves of the soft tissues. Thus, it can be concluded that S100B is usually detected in the neuronal regions.

The value of S100B protein for differentiation of ES from PNES was confirmed in two separate studies (43, 92). S100B works in cell proliferation, migration, apoptosis, and differentiation. Low levels of S100B can induce apoptosis and high level of it can cause cell survival (89). These functions are implicated in brain injuries, and S100B could be used as a brain injury biomarker (89, 92). Previous studies also suggested that the S100B level could be elevated due to mental health problems or a high-stress environment. This elevation of S100B could be detected in the blood because of the increase in blood–brain barrier drainage and elevated ACTH (43). It should be mentioned that the elevation of ACTH in PNES patients had been detected before (42). Further studies are needed to find the role of S100B in PNES patients and also for the confirmation of S100B as a PNES biomarker.

In conclusion, this study aimed to gather the proteins and hormones associated with PNES to clarify the pathomechanism of the disease in different aspects using bioinformatic tools. However, very limited studies have investigated PNES before, and the findings of bioinformatics studies need to be confirmed in experimental research. Therefore, further studies should be done on the molecular and cellular aspects of the disease to explore new genes associated with the disease and confirm our results.

## Data availability statement

The original contributions presented in the study are included in the article/Supplementary material, further inquiries can be directed to the corresponding author.

## Author contributions

MM: study conceptualization, data collection, analysis, and visualization. AJ and SS: data collection and manuscript writing. MK: manuscript writing, revision, and editing. MN: supervision, validation, revision, and editing. All authors contributed to the article and approved the submitted version.

## Conflict of interest

The authors declare that the research was conducted in the absence of any commercial or financial relationships that could be construed as a potential conflict of interest.

## Publisher’s note

All claims expressed in this article are solely those of the authors and do not necessarily represent those of their affiliated organizations, or those of the publisher, the editors and the reviewers. Any product that may be evaluated in this article, or claim that may be made by its manufacturer, is not guaranteed or endorsed by the publisher.

## Supplementary material

The Supplementary material for this article can be found online at: https://www.frontiersin.org/articles/10.3389/fpsyt.2023.1116892/full#supplementary-material

## Footnotes

^1^ DisGeNET.org

^2^ www.ncbi.nlm.nih.gov/clinvar

^3^ https://www.ncbi.nlm.nih.gov/gene

^4^ https://www.orpha.net

^5^ http://stitch.embl.de
